# Supplementary material for: Understanding good communication in ambulance pre-alerts to the emergency department: findings from a qualitative study of UK emergency services
Source: BMJ Open. 2025 Jan 18;15(1):e094221. doi: 10.1136/bmjopen-2024-094221 (PMC11751996; doi:10.1136/bmjopen-2024-094221)
Supplement: online supplemental file 1 [file bmjopen-15-1-s001.docx]

Can you start off by telling me your role and how long you’ve been in the role, and in frontline role/service as a whole

Within this interview I am going to ask you about pre-alert practice in general, but also ask you to reflect on the last pre-alert that you undertook, in order to make it easier to give specific answers.

***Making the decision***

Thinking back to the last patient that you pre-alerted, can you tell us about how you made the decision to pre-alert the patient? (Prompt for factors that affect the decision / whether this was typical / easier / harder than other decisions).

When faced with a difficult pre-alert decision, what do you use to help make the decision? (prompt use of guidance (which?), tools, colleagues)

Can you give examples of difficult pre-alert decisions? What makes an easy decision? What conditions difficult?

Are there any factors that have not already been described that affect how you make a pre-alert decision? (barriers?)

***Undertaking the pre-alert call***

Thinking about the last patient you pre-alerted, can you talk through how you made the call and what information you provided to the receiving ED (prompt – answering questions, use of checklists)

Can you talk to me about how receiving EDs respond to your pre-alert calls? (Prompt – differences, what influences the responses, how does it make them feel? Different between different EDs?)

Can you think of an example of a pre-alert that went well and tell me about why it went well (prompt – certainty of diagnosis, reaction to pre-alert, action at the other end).

Similarly, can you think of a pre-alert that didn’t go well and tell me about that (similar prompts).

***Understanding the value of the pre-alert***

Thinking about the last patient you pre-alerted, what would you have expected the ED to have done in response to your pre-alert call?

Do you think there is anything that you could do to improve how the pre-alert decision is communicated?

Do you think that you pre-alert patients differently now to how you would have done earlier in your career (prompt depending on length of career, 2 yrs ago, 10 yrs ago etc. If so, why? Prompt for changes in understanding, experience of doing pre-alerts)

***Further lessons***

Do you feel that there is clear guidance about which patients get the most benefit from pre-alerts? (why, which ones…)

Do you feel that pre-alerts may be used inappropriately (explore…)

What might help you make pre-alert decisions (prompt guidance about specific decisions)

How can the staff at the receiving ED make the process of undertaking a pre-alert easier?

Is there anything else you’d like to add? Any questions?
