# Supplementary material for: Understanding good communication in ambulance pre-alerts to the emergency department: findings from a qualitative study of UK emergency services
Source: BMJ Open. 2025 Jan 18;15(1):e094221. doi: 10.1136/bmjopen-2024-094221 (PMC11751996; doi:10.1136/bmjopen-2024-094221)
Supplement: online supplemental file 2 [file bmjopen-15-1-s002.docx]

- Can you start off by telling me your role, how long you’ve been in the role and how your role relates to ambulance pre-alerts.
- Thinking back to the last pre-alert call that you dealt with, can you talk me through what happened from when the (red phone) rang (Prompt - tell me about the information you received during the call, understand how they got the information they needed, use of checklists etc.)
- Thinking about the same example, can you tell me what you did in response to the call? (Prompt to understand why they responded like they did, what influenced their decision).
- Can you talk to me about what factors influence how you respond to pre-alerts? (Is this the same for colleagues, has practice changed?) Different responses to pre-alerts or different urgency of pre-alerts. Are all pre-alerts responded to in the same way?
- What do you think is working well about the prealerts system at their particular hospital?
- Are there any ways in which the system is not working well, needs to be improved, etc?
- Are pre-alerts taken seriously, listened to?
- Can you think of examples of useful pre-alerts and how these influenced patient care? (Focus on a specific example, what made it effective) / Can you talk to me about the benefits of pre-alerts (ask for examples).
- Can you give me an example of pre-alerts that were not useful and may have had a negative influence on patient care (Focus on specific example). Can you talk to me about the potential risks of pre-alerts (ask for examples).
- Are there any particular kind of conditions/patients who you feel are pre-alerted too often?
- Are there any particular kind of conditions/patients who you feel should be pre-alerted more than they are currently?
- Do you think there is variation between paramedics in terms of how pre-alert decisions are made?
- What, if anything, do you think the ED could do to make the pre-alert process easier for ambulance clinicians?
- Do you provide feedback to ambulance staff about their pre-alert decisions?
- What, if anything, do you think would help ambulance clinicians to make better pre-alert decisions?

Thank you for talking to us. Is there anything else you’d like to add?
