# Supplementary material for: Understanding good communication in ambulance pre-alerts to the emergency department: findings from a qualitative study of UK emergency services
Source: BMJ Open. 2025 Jan 18;15(1):e094221. doi: 10.1136/bmjopen-2024-094221 (PMC11751996; doi:10.1136/bmjopen-2024-094221)
Supplement: Supplementary file 3 [file bmjopen-2024-094221.reviewer_comments.pdf]

PEER REVIEW HISTORY

BMJ Open publishes all reviews undertaken for accepted manuscripts. Reviewers are asked to complete a checklist review form and are provided with free text boxes to elaborate on their assessment. These free text comments are reproduced below.

ARTICLE DETAILS

Title (Provisional)

Understanding good communication in ambulance pre-alerts to Emergency Department. Findings from a qualitative study of UK emergency services.

Authors

Sampson, Fiona C; O'Hara, Rachel; Long, Jaqui; Coster, Joanne

---

VERSION 1 - REVIEW

**Reviewer**	**1**
**Name**	**Nutbeam, Tim**
**Affiliation**	**Plymouth Hospitals NHS Foundation Trust**
**Date**	**24-Oct-2024**
**COI**	**None**

This is a well-written and valuable paper addressing the critical issue of pre-alert communication between ambulance services and Emergency Departments (EDs). The study provides insightful data on variations in communication and offers recommendations for improving the efficiency of pre-alerts, which are vital for time-critical patient care.

Considerations for Further analysis / exploration:

Exploration of Gender Differences:

Please consider investigating whether gender differences (both of the receiving ED clinicians and the transmitting ambulance clinicians) influence the pre-alert calls. This could be an important aspect to explore in the context of communication effectiveness and interaction.

Call Duration and Word Count:

It would be valuable to report the average duration of pre-alert calls, including the range or standard deviation, and analyse the number of words used. This additional data may help assess the efficiency and conciseness of pre-alert communication.

General Feedback:

Providing some initial context would help frame the significance of pre-alerts more effectively. For instance, including data on how many pre-alerts occur annually and what

proportion of patients they affect could emphasize the scale and importance of improving this communication process.

Be cautious with the first use of acronyms like ETA, ATMIST, and SBAR, ensuring they are spelled out in full. It might also be useful to include supplementary material such as example documents for these forms to support readers unfamiliar with the terms.

Consider expanding the section on technology, highlighting potential improvements like integrated digital pre-alert systems. Additionally, elaborating on the training section with suggestions for incorporating feedback loops, after-action reviews, or joint training and simulations could enhance the paper's practical applications.

The conclusion would benefit from a stronger statement about how these findings could inform national pre-alert standards or policy changes. A couple of additional sentences reinforcing this would underscore the broader relevance of the research.

Reviewer 2

Name Brandenburg, Caitlin

Affiliation Goldcoast Health Service District, Office for Research Governance and Development

Date 26-Oct-2024

COI None

This paper, which qualitatively explores the use of ambulance pre-alerts, is robustly conducted and appropriately reported. The authors should be commended for their work in this little-researched yet important space. The findings have been presented in a practical way which can directly guide real-world improvements. The paper is of high quality overall, though there are some areas for improvement, particularly in the methods, which I outline in detail below.

Introduction:

"There is evidence of variation in the quality of information provided within pre-alerts, with high proportions of pre-alerts providing inaccurate, unnecessary or inadequate information to enable the EDs to appropriately mobilise resources. (12-14)" As this is the crux of the impetus for this study, please give some more detail about the findings of these three studies. Perhaps bring out into a separate paragraph which also includes the sentences on findings of 16 and 17.

"Despite the increasing body of evidence supporting the use of pre-alerts for patients requiring time-critical treatment, there is a lack of research exploring how pre-alerts should be communicated to maximise effectiveness. (15) Brunson L, Lauzier-Jobin F, Olson B, Côté L-

P. Seven key insights from critical realism and their implications for ecological thinking and action in community psychology. Journal of Community Psychology. n/a(n/a)." It is unclear how reference 15 relates to this sentence? Please clarify

Methods:

Please give some brief detail about the overarching mixed methods project, to put this study in context.

Please give the qualitative approach used, as per SRQR- "Qualitative approach (e.g., ethnography, grounded theory, case study, phenomenology, narrative research)". It seems as if a descriptive qualitative approach may fit best.

"Ambulance clinicians were initially selected purposively, (21)sampling for length of experience, sex, role, ethnicity and whether they were high or low pre-alerters according to routine data. We recruited selected clinicians from direct invite before expanding recruitment via open invitations facilitated by research leads at each ambulance service. We also interviewed clinicians who we identified during observations at individual EDs"- it seems as if a combination of purposive and convenience sampling has been used? If so, please state up front, otherwise it may be unclear what is meant by "initially".

"non-participant observations"- I'm not familiar with this terminology, does this mean observations of people who weren't consenting participants? Could this be made clearer?

"Data collection continued until the minimum proposed number of interviews were undertaken and thematic saturation occurred during analysis."- what was the minimum proposed number of interviews? Was this site by site? Was there an element of quota sampling?

"Interviews were conducted remotely, either online or by phone"- is this true even of participants who were identified during site observations?

"Interview topic guides were developed in collaboration with the project management and PPI group."- were these piloted? Were they refined after the first few interviews or stable throughout the study?

"The field researchers (JL and JC) had no prior experience and had fewer pre-conceptions about how emergency services worked than ROH and FS."- it may also be relevant, if the researchers are comfortable, to note whether the researchers have a lived experience of pre-alert as a patient or family member.

"Braun & Clark"- should be Clarke

It is unclear from the data analysis section how observation data fed into analysis, as only interviews are mentioned.

Results

Themes are well-described and have clear titles. Each theme has been well-supported by participant quotes. In order to aid the reader, authors could consider providing a list of the themes before describing each one in detail, for example by bullet points in text, or a table of theme names. This may help orient the reader to the overall findings.

Theme 1: "individual clinician factors"- these factors seem to be seniority and experience, so I wonder if the theme name could be more specific

In general, there seems to be a crosscutting theme about differences in preferences between locations, and between individual clinicians, with this variability causing difficulties for the paramedics in meeting expectations for a "good" pre-alert call. Authors may consider whether this is a separate theme.

Discussion

The discussion is appropriate and covers key findings with reference to the literature, particularly what can be learnt from handover process improvements. It also provides a set of practical implications for ED and ambulance services to consider in their own context.

VERSION 1 - AUTHOR RESPONSE

Comment	Response
**Reviewer 1**	
General comment	Thank you
Please consider investigating whether gender differences (both of the receiving ED clinicians and the transmitting ambulance clinicians) influence the pre-alert calls. This could be an important aspect to explore in the context of communication effectiveness and interaction.	We did not specifically explore whether the gender of receiving ED clinicians and the transmitting ambulance clinicians influenced the pre-alert call. During observations we were sometimes unaware of the gender of the transmitting ambulance clinician and it was not appropriate to ask for this information during a time critical emergency. Gender did not come up as a sub-theme during our analysis of interview data, however seniority did and this is sometimes linked to gender with more male senior staff. However, we do not think that it is appropriate to include gender differences in this paper as it was not specifically raised by any of the 74 interviewees. Interestingly we did consider whether the gender of the patient was a factor in whether they receive a pre-alert in our analysis of pre-alert and hospital routine data, and we identified that male patients were more likely to be pre-alerted than female patients.
It would be valuable to report the	It was not possible to systematically record this

average duration of pre-alert calls, including the range or standard deviation, and analyse the number of words used. This additional data may help assess the efficiency and conciseness of pre-alert communication.	information during the observations. The layout of each ED and resus area varied, there were between 1 and 3 pre-alert phones in each ED, with some EDs having pre-alert phones in majors and in resus. On some occasions multiple pre-alert calls happened at the same time and this could also be at the same time as a pre-alert was being brought in. Whilst we had 2 researchers undertaking observations at busier sites, our focus was on the response to the pre-alert call rather than its duration. We agree that content analysis of the recorded calls would be useful in helping to assess the efficiency and conciseness of pre-alert communication, but this would need to be facilitated by pre-alert calls taking place on recorded lines, which in the majority of cases they were not.
Providing some initial context would help frame the significance of pre-alerts more effectively. For instance, including data on how many pre-alerts occur annually and what proportion of patients they affect could emphasize the scale and importance of improving this communication process.	We have added the following to the final sentence of the first paragraph in the background section to emphasise the scale and frequency of pre-alerts - Pre-alerts are increasingly a key component of patient pathways for a range of conditions such as stroke, STEMI and major trauma (5-8) **and in England >1 in 10 ambulance conveyances are pre-alerts.** (reference Sampson, F., et al., *PP33 Which patients receive a prealert? Analysis of linked data in three ambulance services.* Emergency Medicine Journal, 2023. **40**(Suppl 1): p. A14-A14.)<br><br>We have also submitted an article based on the above conference abstract as a separate paper to BMJ Open (bmjopen-2024-097122)
Be cautious with the first use of acronyms like ETA, ATMIST, and SBAR, ensuring they are spelled out in full. It might also be useful to include supplementary material such as example documents for these forms to support readers unfamiliar with the terms.	These terms are now all spelled out in full the first time they are used.
Consider expanding the section on technology, highlighting potential improvements like integrated digital pre-alert systems. Additionally, elaborating on the training section with suggestions for incorporating feedback loops, after-action reviews, or joint training and simulations could enhance the paper’s practical applications.	We have reviewed the coded data relating to this following this comment. However, these suggestions did not come up in the interviews, therefore we cannot include this in the findings.

The conclusion would benefit from a stronger statement about how these findings could inform national pre-alert standards or policy changes. A couple of additional sentences reinforcing this would underscore the broader relevance of the research.	A sentence has been added at the end of the implications section of the discussion which states “The findings from this research are being used to inform revision of national pre-alert policy.”
**Reviewer 2**	
General	Thank you
“There is evidence of variation in the quality of information provided within pre-alerts, with high proportions of pre-alerts providing inaccurate, unnecessary or inadequate information to enable the EDs to appropriately mobilise resources. (12-14)” As this is the crux of the impetus for this study, please give some more detail about the findings of these three studies. Perhaps bring out into a separate paragraph which also includes the sentences on findings of 16 and 17.	References 12 – 14 are >15 years old therefore we do not want to put too much emphasis on these in the paper. We have clarified this in the paper by amending the sentence to “There is a lack of evidence around pre-alerts, however some evidence from the early 2000s describes variation in the quality of information provided within pre-alerts, with high proportions of pre-alerts providing inaccurate, unnecessary or inadequate information to enable the EDs to appropriately mobilise resources. (12-14)”<br><br>References 16 and 17 relate to findings from our mixed method study. We have revised this paragraph to ensure this is clearer within the text “As part of our wider mixed-methods exploring the use of pre-alerts, we conducted a survey of UK ambulance clinicians, which identified significant differences in the use of mnemonics or structured formats during pre-alert. (17) In the present study, we aimed to explore pre-alert communication using our qualitative data to understand how pre-alert information is communicated and how communication can be improved to optimise the ED response for pre-alerted patients.”
“Despite the increasing body of evidence supporting the use of pre-alerts for patients requiring time-critical treatment, there is a lack of research exploring how pre-alerts should be communicated to maximise effectiveness. (15) Brunson L, Lauzier-Jobin F, Olson B, Côté L-P. Seven key insights from critical realism and their implications for ecological thinking and action in community psychology. Journal of Community Psychology. n/a(n/a).” It is unclear how reference 15 relates	This was incorrectly referenced and has been corrected.

to this sentence? Please clarify	
Please give some brief detail about the overarching mixed methods project, to put this study in context.	Additional information has been included at the start of the methods section to provide this context.
Please give the qualitative approach used, as per SRQR- “Qualitative approach (e.g., ethnography, grounded theory, case study, phenomenology, narrative research)”. It seems as if a descriptive qualitative approach may fit best.	We have added the information that this was a descriptive qualitative approach.
“Ambulance clinicians were initially selected purposively, (21)sampling for length of experience, sex, role, ethnicity and whether they were high or low pre-alerters according to routine data. We recruited selected clinicians from direct invite before expanding recruitment via open invitations facilitated by research leads at each ambulance service. We also interviewed clinicians who we identified during observations at individual EDs”- it seems as if a combination of purposive and convenience sampling has been used? If so, please state up front, otherwise it may be unclear what is meant by “initially”.	This section has been reworded to clarify the sampling process
“non-participant observations”- I’m not familiar with this terminology, does this mean observations of people who weren’t consenting participants? Could this be made clearer?	This term has been removed and further information provided to clarify that the researchers were non-clinicians and not participating in the work of the ED.
“Data collection continued until the minimum proposed number of interviews were undertaken and thematic saturation occurred during analysis.”- what was the minimum proposed number of interviews? Was this site by site? Was there an element of quota sampling?	Information regarding proposed numbers has been added, also noting that the range of roles as described in the sampling strategy section was of more significance.
“Interviews were conducted remotely, either online or by phone”- is this true even of participants who were identified during site observations?	All interviews were conducted remotely – participants were too busy whilst on shift to conduct interviews during the observations.

"Interview topic guides were developed in collaboration with the project management and PPI group."- were these piloted? Were they refined after the first few interviews or stable throughout the study?	The topic guides were not piloted. We have provided more information within the paper on the refinement of the topic guide after the first few interviews.
"The field researchers (JL and JC) had no prior experience and had fewer pre-conceptions about how emergency services worked than ROH and FS."- it may also be relevant, if the researchers are comfortable, to note whether the researchers have a lived experience of pre-alert as a patient or family member.	This sentence has been changed to clarify this point.
"Braun & Clark"- should be Clarke	This has been amended.
It is unclear from the data analysis section how observation data fed into analysis, as only interviews are mentioned.	When we undertook observation sessions at each ED we also used this as a recruitment opportunity for the qualitative interviews with ED clinicians, in terms of identifying the key people to interview and talking to them about taking part in an interview. The observations also informed our knowledge and understanding of pre-alert practice at each ED site and the variation in responses. We were unable to record direct quotes during observations, however we made detailed notes which were uploaded to Nvivo and used to understand the context of each interview from the site. The observations also identified topics that we wanted to ask about in the interviews. Whilst we primarily quote from or refer to the interviews within the analysis, the interviews are reflective of what we observed and provide data that is more amenable to presentation in a paper.
Themes are well-described and have clear titles. Each theme has been well-supported by participant quotes. In order to aid the reader, authors could consider providing a list of the themes before describing each one in detail, for example by bullet points in text, or a table of theme names. This may help orient the reader to the overall findings.	Thank you for your comments. We have added a Table 4 containing a list of the themes at the start of the findings to help.
Theme 1: "individual clinician factors"- these factors seem to be seniority and experience, so I wonder if the theme name could be	These two factors - seniority and experience – have been added to the theme title

more specific	
In general, there seems to be a crosscutting theme about differences in preferences between locations, and between individual clinicians, with this variability causing difficulties for the paramedics in meeting expectations for a “good” pre-alert call. Authors may consider whether this is a separate theme.	We have considered the reviewers comment about differences in preferences between locations and individual clinicians causing difficulties in meeting expectations for a ‘good pre-alert call’. We feel that the overriding issues here are the lack of guidance and training, which could help to improve shared expectations and consistency between locations and clinicians. Therefore we have not included this as a separate theme.
The discussion is appropriate and covers key findings with reference to the literature, particularly what can be learnt from handover process improvements. It also provides a set of practical implications for ED and ambulance services to consider in their own context.	Thank you.
